# Using *Paramecium* as a Model for Ciliopathies

**DOI:** 10.3390/genes12101493

**Published:** 2021-09-24

**Authors:** Megan Valentine, Judith Van Houten

**Affiliations:** 1 State University of New York at Plattsburgh, 101 Broad Street, Plattsburgh, NY 12901, USA; mvale016@plattsburgh.edu; 2 Department of Biology, University of Vermont, 120 Marsh Life Science, 109 Carrigan Drive, Burlington, VT 05405, USA

**Keywords:** *Paramecium*, cilia, ciliate, ciliopathy

## Abstract

*Paramecium* has served as a model organism for the studies of many aspects of genetics and cell biology: non-Mendelian inheritance, genome duplication, genome rearrangements, and exocytosis, to name a few. However, the large number and patterning of cilia that cover its surface have inspired extraordinary ultrastructural work. Its swimming patterns inspired exquisite electrophysiological studies that led to a description of the bioelectric control of ciliary motion. A genetic dissection of swimming behavior moved the field toward the genes and gene products underlying ciliary function. With the advent of molecular technologies, it became clear that there was not only great conservation of ciliary structure but also of the genes coding for ciliary structure and function. It is this conservation and the legacy of past research that allow us to use *Paramecium* as a model for cilia and ciliary diseases called ciliopathies. However, there would be no compelling reason to study *Paramecium* as this model if there were no new insights into cilia and ciliopathies to be gained. In this review, we present studies that we believe will do this. For example, while the literature continues to state that immotile cilia are sensory and motile cilia are not, we will provide evidence that *Paramecium* cilia are clearly sensory. Other examples show that while a *Paramecium* protein is highly conserved it takes a different interacting partner or conducts a different ion than expected. Perhaps these exceptions will provoke new ideas about mammalian systems.

## 1. Introduction

Microscopists in the 1600s were captivated by *Paramecium* species swimming behavior when examining infusoria [1]. John Hill writes in 1720 in his *History of Animals* that Paramecia are easy to see with “a third magnifier in the double microscope.” He notes that these little animals are very swift in motion, can twist and turn about its axis and even fold up [2]. From the very beginning of observations of *Paramecium*, motility was their hallmark. These early microscopists did not correctly know how the paramecia were moving in their watery surrounds (see [3] for a succinct history of the study cilia and ciliary disease).

**Figure 1:**
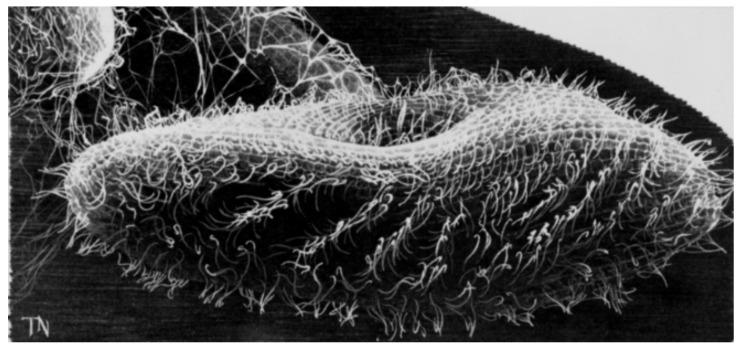
Line drawing from a scanning electron microscope image of *P. tetraurelia.* Courtesy of J. Van Houten and 1988 Grass Calendar.

We know now that paramecia are propelled by thousands of motile cilia (Figure 1), which are long (10 µm), thin, membrane-covered organelles that protrude from the cell surface. They move in a beautiful, physically constrained synchrony called metachronal waves that keep them from tangling or interfering with one another (Figure 1). A comparison is usually made to a field of grain swaying in waves in the wind. Special motor dyneins move the cilia in graceful arcs that have a power stroke and lazy return stroke (see [4] for a review). The power stroke toward the posterior causes the cell to swim forward and toward the anterior (usually just transiently) causes a brief turn. This behavior caught the attention of the original microscopists as they observed infusoria, and will be explained in more detail below because the internal mechanisms and electrical controls are highly conserved.

**Figure 2:**
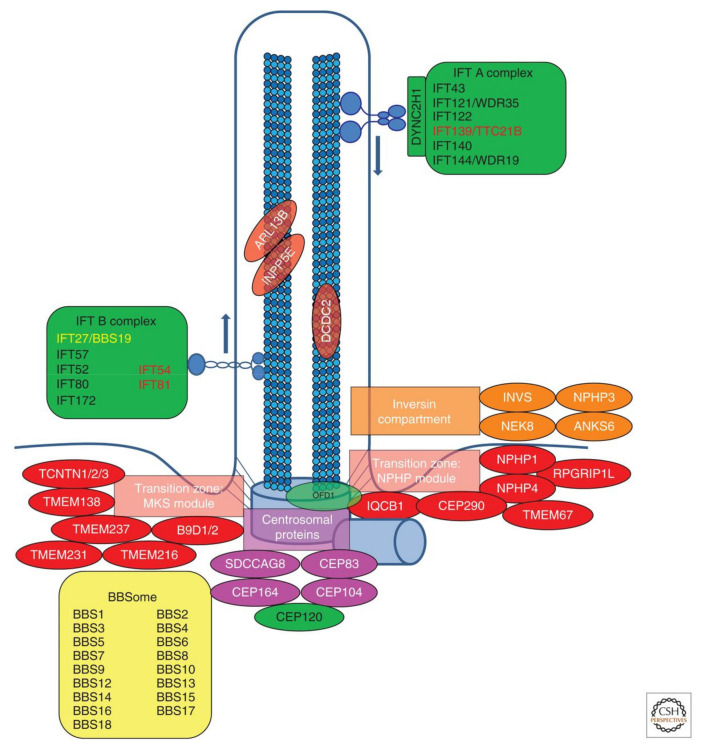
Subcellular localization of protein encoded by monogenic genes of nephronophthisis-related cilioipathies (NPHP-RC). Subcellular localization of proteins encoded by monogenic genes of NPHP-RC is depicted. Proteins are color-coded based on their respective disease group: Nephronphthisis (NPHP), Senior–Loken syndrome (SLS), Joubert syndrome (JBTS), Meckel–Gruber syndrome (MKS), Bardet–Biedl syndrome (BBS), and skeletal ciliopathies. It becomes apparent that disease groups cluster to distinct subcellular localizations. IFT, intraflagellar transport. Reproduced from [5], with permission.

**Figure 3:**
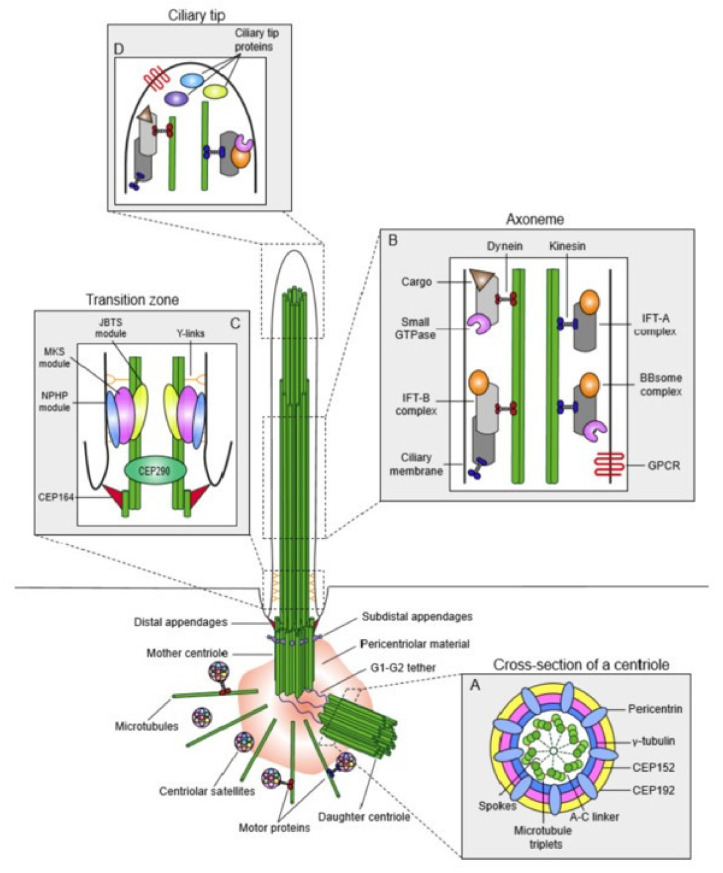
Overview of the anatomy of the centrosome/cilium complex and its sub-compartments. This complex is composed of three compartments, the centriole, the cilium, and the centriolar satellites. In primary cilia, the centrosome, consisting of two microtubule-organizing centers, recruits pericentriolar material (PCM). In Paramecium, these structures are not present, but instead each cilium is anchored by a basal body. In both, the centrioles (or basal bodies) differ in age where one is called the mother (the older) and the younger, called the daughter. The mother has distal appendages, and for primary cilia, the mother also acts as the basal body for cilium assembly. The primary cilium itself is compartmentalized into different regions, including the transition zone (TZ), the axoneme, and the ciliary tip. (**A**) A cross section of the proximal end of the centrioles containing nine triplets of microtubules symmetrically arranged in a ring connected by A–C linkers. These triplets are connected to the inner core of the centriole by radial spokes. The PCM material is organized into concentric layers and those layers are overlayed by a filamentous material, pericentrin. (**B**) The transition zone (TZ) has microtubule pairs, as the outmost microtubule does not extend the length of the cilium. This region acts as a barrier that regulates proteins into and out of the cilium through the NPHP-MKS-JBTS module. Transition fibers help to anchor the basal body to the ciliary membrane. (**C**) The ciliary axoneme is the core of the primary cilium and lacks a central pair of microtubules (that are present in motile cilia). These outer doublets of microtubules act as roadways for the IFT-A and IFT-B complexes, as well as the BBSome, to move cargo along the cilium. IFT-B and anterograde transport relies on kinesin-2 (blue) motors while retrograde transport and IFT-A rely on cytoplasmic dynein-2 (red) motors. The BBSome complex interacts with the IFT particles the move cargo into and out of the cilium. (**D**) The ciliary tip is a specialized region of primary cilia. Here, IFT particles, Hedgehog pathway components, and other microtubule-associated proteins work to regulate IFT remodeling, the length of the cilium, and Hedgehog signaling. Reproduced from [19], with permission through CC BY license.

**Figure 4:**
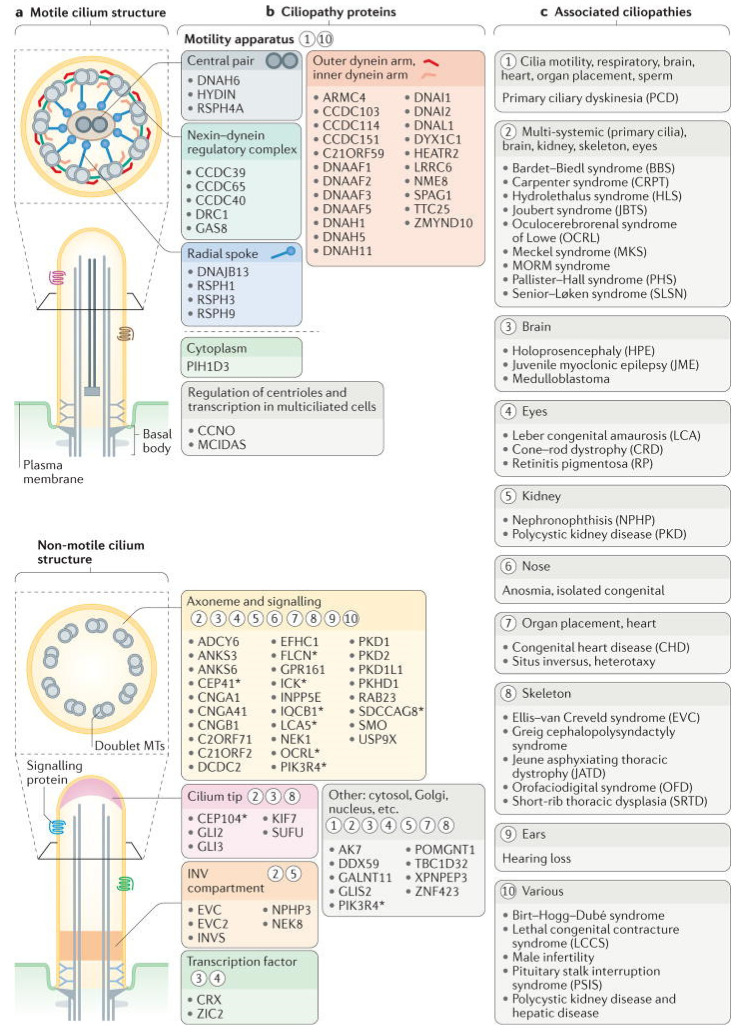
Structural and functional features of motile and sensory cilia are associated with ciliopathies. (**a**) the major structures of motile and non-motile cilia. (**b**) Major sites of action for ciliopathy-associated proteins that are components of motile cilia (motility apparatus or transcription factors required for the generation of motile cilia) and sensory cilia (axonemal and signaling proteins, ciliary tip proteins or inversin (INV) compartment proteins). The asterisks indicate proteins that are also localized to other ciliary regions during ciliogenesis or ciliary trafficking. Circled numbers indicate one or more ciliopathies that result from defects in the different ciliary compartments and proteins. (**c**) Ciliopathies grouped into major categories that are associated with proteins and ciliary regions in part B. Reproduced from [7], with permission.

We focus here on the use of *Paramecium* as a model organism for the study of cilia and insights into ciliopathies, which are human diseases caused by defects in ciliary structure or function [3,5,6]. Sometimes these diseases can be traced back to inherited gene defects, but all are associated with the failure of cilia to carry out their movement or sensory functions. Modern medical genomics studies have shown us that there are a myriad of genes and many syndromes associated with ciliopathies [5,7]. See Figure 2 and Table 1 in [5] and Figure 3, Figure 4 and Figure 5 in [7] for a sense of their range.

**Table 1:** Ciliopathy Genes of *Paramecium*.

	Name	Alias/Other Names	Present in *Paramecium*?	Reference, if Examined in *Paramecium*
BBS Module	BBS1		YES	[8]
	BBS2		YES	[8]
	BBS3	ARL6	YES	[8]
	BBS4		YES	[8]
	BBS5		YES	[8]
	BBS6		NO	--
	BBS7		YES	[8]
	BBS8	TTC8	YES	[8]
	BBS9		YES	[8]
	BBS10	C12orf58	NO	--
	BBS11	TRIM32	NO	--
	BBS12	C4orf24	NO	--
	BBS15	WDPCP	NO	--
	BBS17	LZTFL1	NO	--
	BBS18	BBIP1	NO	--
	BBS19	IFT27/RABL4	YES	[9]
MKS Module	MKS1	BBS13	YES	[10]
	MKS2	TMEM216	YES	[11]
	MKS3	TMEM67 or Meckelin	YES	[12]
	Ahi1	JBTS3 or Jouberin	YES	
	B9D1	MKS9	YES	
	B9D2	MKS10	YES	[13]
	Tectonics (1,2,3)	TCTN1, TCTN2, and TCTN3	YES	
	TMEM17		YES	
	TMEM107		YES	[11]
	TMEM218		NO	--
	TMEM231	JBTS20, OFD3, or MKS11	YES	
	TMEM237	JBTS14	NO	--
NPHP Module	NPHP1		NO	--
	NPHP4	POC10	YES	[11]
	NPHP5	IQCB1	NO	--
Others	NPHP3	MKS7	NO	--
	CEP290	NPHP6, MKS4, BBS14	YES	[11]
	RPGRIP1L	NPHP8 or MKS5	YES	[11,13]
	TTC12		YES	[14]
	C11orf70		YES	[15]
IFT B Complex *	IFT38/40	Cluap1, qilin, FAP22	YES	[16]
	IFT46		YES	[9]
	IFT57		YES	[17]
	IFT80		YES	[9]
	IFT81			
	IFT88	Tg737	YES	[12,17]
	IFT172		YES	[9]
IFTA Complex *	IFT139		YES	[9]
	IFT140		YES	[18]

Abbreviations: ARL6, ADP Ribosylation Factor Like GTPase 6; BBIP1, BBSome Interacting Partner 1; BBS, Bardet-Biedl Syndrome; CEP290, Centrosomal protein of 290 kD; IFT, Intraflagellar Transport; IQCB1, IQ Motif Containing B1; JBTS, Joubert Syndrome; LZTFL1, Leucine Zipper Transcription Factor Lake-1; MKS; Meckel-Gruber Syndrome; NPHP, Nephronophtysis; RPGRIP1L, Retinitis pigmentosa GTPase Regulator-Interacting Protein 1-Like Protein; TCTN, tectonic proteins 1, 2, and 3; TMEM, Transmembrane protein; TRIM32, Tripartite Motif Containing 32; TTC, Tetracopeptide Repeat Domain; TZ, Transition zone; WDPCP, WD Repeat Containing Planar Cell Polarity Effector. * Only those Intraflagellar Transport Proteins that have been examined in *Paramecium* are shown here.

**Figure 5:**
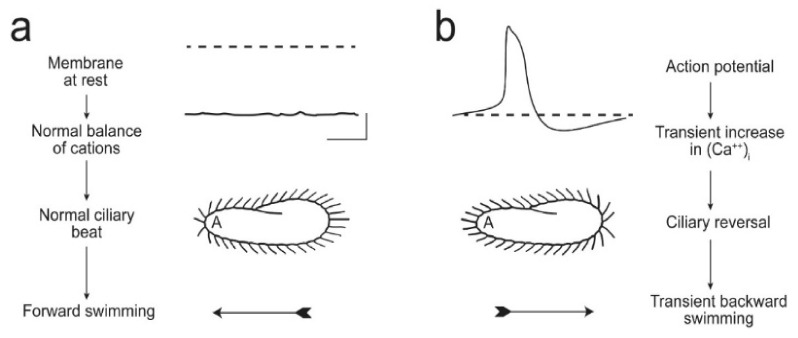
These images illustrate (**a**) that the resting membrane potential of *Paramecium* is negative; the ciliary beat is toward the posterior and cell swims forward. (**b**) In depolarizing conditions, such as high K or Ba solutions, the cell’s membrane depolarizes and reaches threshold for the action potential, during which Ca^2+^ enters the cilium through Ca_V_ channels and the Ca^2+^ changes the power stroke toward the anterior, moving the cell backward. The action potential is quickly terminated, returned to resting V_m_ levels, and the extra Ca^2+^ removed. Reproduced from [20] with permission.

Failure of motile cilia to move mucous, spinal fluid, sperm, and fluid of the embryonic node results in Primary Ciliary Dyskinesia (PCD) or Kartagener Syndrome [5,6].

Motile cilia clear mucous from the respiratory tract, where, if left in place, would lead to pneumonia and destruction of the respiratory tissues. Other symptoms include *sinus inversus*, congenital heart disease, infertility, and more. *Paramecium* with its thousands of motile cilia can provide some insights into PCD but also into syndromes caused by failure of non-motile cilia (called primary cilia) to signal and function. Defects in primary cilia structure or function results in polycystic kidney disease (PKD) with enlarged kidneys and massive cysts or nephronophthisis (NPHP) with small and fibrotic kidneys and cysts. Other potential abnormalities can occur in many organ systems such as retinal degeneration, skeletal abnormalities, hepatic fibrosis, brain malformation, and more. Whether motile or non-motile, cilia are sensory organelles with important functions to fulfill in human development [21,22,23]. 

There are other critical players in the formation of motile and primary cilia, such as the Bardet-Biedl syndrome (BBS) proteins, which form a cargo adapter to bring membrane and signaling proteins to the apparatus that moves them to and into the cilia, the Intraflagellar Transport (IFT) complexes [24,25]. Failure of this BBSome to function properly leads to a constellation of symptoms of retinal degeneration, cystic kidneys, short fingers and genitalia, and more.

An intense new interest in cilia, in large part brought about the identification of the connection of PKD to flagellar transport [26], quickly led to the identification of genes and proteins associated with ciliary diseases [3]. These proteins that compose, build, and carry out the sensory functions of cilia could be organized into “biochemically and functionally distinct” modules in common to a disease, location in the cilia and cytoplasm, or role in cilia formation or function [27].

Figure 2 shows a relatively recent rendering of the organization of ciliopathy genes products and modules [5]. Figure 3 shows the outcome of proximity mapping studies of many of the gene products shows that they could be assigned to modules [19]. However, while the gene products overlap in location or function (Figure 4) they do not always form a larger physical complex such as the BBSome or the Intraflagellar Transport (IFT) A or B complexes [27,28,29,30,31,32,33]. 

Braun and Hildebrandt [5] in Figure 2 organize their collection of ciliopathy genes somewhat differently from Arslanhan’s [19] (Figure 3) and from Reiter and Leroux’s (Figure 4) [7] arrangements in their reviews. Nonetheless, there are common themes of motile cilia vs. immotile sensory ciliopathies, and both provide refreshers of the structure of cilia. 

We have organized this overview differently from a comprehensive review of the structure and function of *Paramecium* cilia. Instead, we have organized by ciliopathy and have tried to point out the insights from these cilia, which are motile, for ciliopathies including some caused by defects in primary immotile cilia (see Table 1). We propose to add to this current understanding through the use of *Paramecium* examples of cilia to bring a broader understanding of function. At this juncture, we would like to stress that *Paramecium* research can provide these insights because of the bounty of material in the large number of cilia per cell and the legacy of many talented researchers working with *Paramecium*. Moreover, the arrangement of cilia and basal bodies on the cell surface makes it glaringly obvious when a mutation has interfered with this pattern, and easily observable swimming behavior governed by ion channel activity allows *Paramecium* cells to “broadcast” the normal or compromised function of these channels [34]. (Our two labs are invested in this research and below you will find us referring to “we”, meaning the Van Houten and/or Valentine labs. Unpublished research has been labeled.)

Some of the research techniques the reader will encounter below are listed in Table 2.

## 2. BBS Proteins

Bardet-Biedl Syndrome (BBS) is associated with fourteen or more genes and is characterized by multiple symptoms that are from the failure of cilia to signal: obesity, hypogonadism, polydactyly, retinal degeneration, mental retardation, and kidney cysts [35]. Seven of the BBS protein products (BBS 1,2,4,5,7,8,9) with BB1P10 (also called BBS18) form the BBSome [36]. With small GTPases, the BBSome assembles into a coat that traffics Golgi vesicles with their valuable cargo to the IFT apparatus where some of the cargo is inserted into the ciliary membrane [36,37,38,39]. G protein coupled protein receptors, such as somatostatin receptor 3 [37] and neuropeptide Y [40], depend upon the BBSome for ciliary localization while other proteins like the signaling protein Phospholipase D depend upon the function of the BBSome to exit the cilium [24,25,41]. Overall, the BBSome has a lot of sway over the ciliary membrane proteome.

*Paramecium tetraurelia* has orthologs of the human BBS genes [8]. For this study, we established a collaboration between the Van Houten and Cohen labs. To examine the protein products of these genes, we FLAG-tagged BBS8 and BBS9 for immunoprecipitation. In the precipitates, we found BBS1,2,4,5,7,8,9. BBS3 that is not part of the human BBSome was not found in the *P. tetraurelia* precipitates while a homolog for BBS3 exists. Therefore, we considered that, in *P. tetraurelia*, BBS proteins probably interact in a large complex as in human cells. 

RNAi for all *Paramecium* BBS genes except 7 and 9 showed no change in ciliary number or length. However, RNAi for BBS 7 or 9 led to bald patches on the cell surface and shorter cilia among those few remaining.

We showed that RNAi depletion of BBS gene products leads to loss of ciliary K channels and another ciliopathy channel (PKD2), but not other ion channels and sensory proteins from the cilia. To understand the roles and significance of these channels, we will take a short diversion through *Paramecium* physiology, ciliary beating, and swimming behavior. 

The Figure 5 below summarizes many decades of work by superb electrophysiologists and behavioral biologists. In Figure 5a, shows a *Paramecium* cell swimming forward with cilia beating the power stroke toward the posterior concurrent with a negative membrane potential. The next scenario in Figure 5b shows the cell with ciliary power stroke reversed toward the anterior, and the membrane potential showing an action potential. Because of that action potential, the cell transiently reverses course, and when it resumes, it usually has changed swimming direction. In Figure 5b, a strong depolarization of the cell (from bumping into an object, the touch of a predator on the anterior, high salt environment, or repellent chemical cues) leads to regenerative Ca action potential, with Ca^2+^ entering through voltage-gated channels that are *exclusively in the cilia* [42,43]. This increased intraciliary Ca^2+^ interacts with the axoneme and reverses the power stroke. 

**Figure 6:**
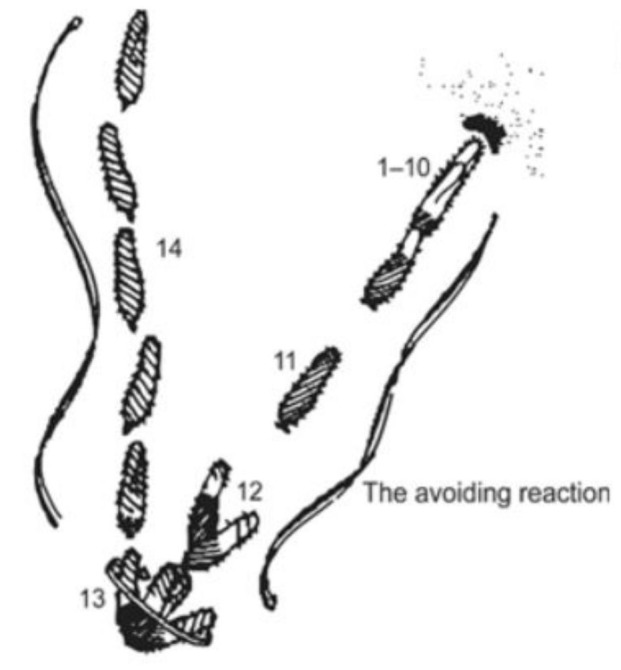
Series of steps in the swimming path of a *Paramecium* cell that bumps into a solid object (upper right), reverses, pivots in place, and finally swims off in a new direction [44].

Jennings [44,45] famously described these turns in swimming as avoiding reactions (Figure 6). Jennings’ careful observations of swimming behavior interested very talented physiologists, Eckert, Machemer, Naitoh, Kaneko, and later others, who used electrophysiology to show that motion of *P. caudatum* is controlled by ion conductances [46,47,48]. Hence, *Paramecium* became known as a little swimming neuron as these physiologists came to show us that the control of forward and backward swimming comes from the ion channels of the ciliary membrane.

A side note on the usefulness of the *Paramecium* cilia is that the cells can be deciliated and the cilia will regrow on the cell even while impaled on an electrode [42,43]. This procedure allows us to monitor the return of specific channels or sensory proteins using electrophysiology. Therefore, in addition to showing us that the voltage-gated Ca_V_ were exclusively on the cilia, Dunlap [42] was able to show that cilia must regrow more than half of their length before the action potentials return, suggesting that these channels are primarily on the distal end of the cilium.

The action potential ends and the membrane potential repolarizes to rest by Ca^2+^ feeding back to inactivate the Ca_V_ channel and, separately, activates two types of hyperpolarizing K channels [48,49]. The depolarization phase of the action potential itself activates the voltage dependent ciliary K channel (K_V_) and the Ca^2+^ that enters the cilium through the Ca_V_ channels during the action potential more slowly activates the calcium-dependent ciliary K channel (K_Ca_) [50,51]. 

We first knew from physiology that members of these two types of K channels, like the voltage-gated Ca channels, seem to be concentrated in the ciliary membrane and absent from the soma membrane [46,48]. K channel genes were found by Haynes and Kung to be highly abundant (perhaps 800 or more) [52], hindering the search for ciliary K channel proteins activated by the action potential. However, LC-MS/MS helped us in the Van Houten lab in the next stage to identify specific K_Ca_ channels unique to the ciliary membrane [53]. Additionally, Yano tagged and followed one of these many channels (SK1a) where it was visualized in the cilia and appeared to be absent from the cell body membrane [8].

Using genomics and LC-MS/MS, we also identified three Ca_V_ channel proteins unique to the ciliary membrane. Expression of these proteins in tagged form by Yano and Lodh was a tour de force given their large size (~250 kd), but this helped us to confirm their location in the cilia [53,54,55].

We asked whether these three Ca_V_ and SK1a channels and some other ciliary membrane proteins rely upon the BBSome to enter or remain in cilia. To monitor these channels, we (Van Houten and workers) used two methods, epitope tagging described below and behavioral tests described here. The duration of backward swimming is an indicator of Ca^2+^ in the cilia because, as described above, Ca^2+^ enters through the Ca_V_ and causes backward swimming. If the mechanisms for repolarization after the action potential, i.e., the K_V_ and K_Ca_ channels, do not function properly, cells will remain depolarized and swim backward longer. We can distinguish between the activity of the K_V_ and K_Ca_ channels to determine which of these kinds of channels might have failed: 30 mK KCl is used to examine the function of K_V_ and tetraethylammonium solutions inhibit the K_V_ leaving the K_Ca_ channels to do the job of repolarizing. We found that after strong depolarization, cells with their *BBS 7 or 9* genes silenced lost K_Ca_ channel function, those with their *BBS 5* gene silenced lost the function of K_V_ channels, while those with their *BBS3* gene silenced lost the function of both kinds of K channels of the cilia. Therefore, both types of ciliary K channels relied upon the BBSome and BBS3for trafficking but required different BBS proteins [8].

**Figure 7:**
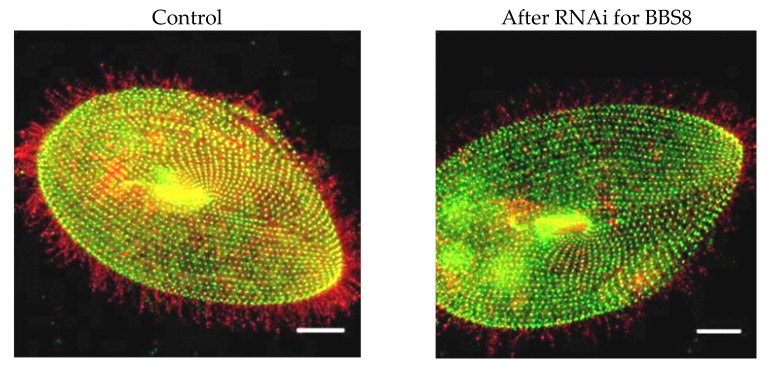
Immunofluorescence of FLAG-Xnt, an interacting partner of PKD2. Only the merged images are shown here. FLAG control is a cell microinjected with FLAG-pPXV vector and fed RNAi empty vector bacteria (not shown). FLAG-Xnt controls are cells expressing FLAG-Xnt and fed bacteria with an RNAi empty vector. “BBS8”cells are expressing FLAG-Xnt and are also depleted of BBS8 mRNA by RNAi. Cells were immunostained with anti-FLAG (red) and basal body (green for *Tetrahymena* centrin) antibodies. (Valentine unpublished).

**Figure 8:**
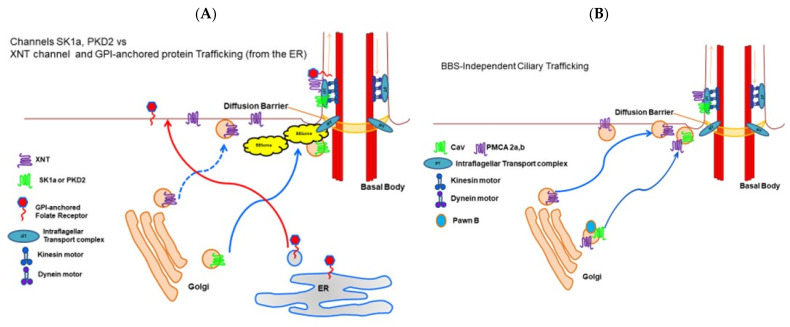
Summary cartoons of trafficking of membrane proteins to the cilia. (**A**) shows the folate receptor with its fatty acid tail moving from the ER directly to the plasma membrane before diffusing into the ciliary membrane. The XNT protein emerges from the Golgi on vesicles and moves to the plasma membrane and then to the contiguous ciliary membrane. The protein channels SK1a and PKD2 also emerge from the Golgi on vesicles but these vesicles are guided by the BBSome past the TZ and into the ciliary membrane. The motor proteins dynein and kinesin move the IFT complexes up and down the cilium and deposit their cargoes in the cilium. (**B**) shows that the calcium channel and its putative partner the plasma membrane calcium pump move on vesicles from the Golgi to the ciliary membrane without aid of the BBSome. When they reach the ciliary membrane, they are transported past the TZ and into the cilium.

In a different second approach to examining the presence of channels and sensory proteins in cilia, we epitope-tagged the SK1a K_Ca_ channel, Ca_V_1 channel, PKD2 channel and its partner XNT channel [56] and used a specific antibody to visualize a glycosylphosphatidyl inositol anchored folate chemoreceptor. By following these proteins for their fluorescence tags, we confirmed that the K_Ca_ channel and PKD2 require the function of the BBSome proteins BBS7, 8, and 9 [8], but XNT did not (Figure 7 unpublished data). Interestingly, other channels and proteins of the cilia also do not depend upon the BBSome for ciliary membrane localization: The Ca_V_ channels and the folate chemoreceptor reach the cilia without the BBSome [8]. For a summary, see Figure 8A,B.

While Ca^2+^ enters cilia through the dedicated Ca_V_ channels, this Ca^2+^ must be sequestered or removed to prepare the channels for the next action potential because Ca^2+^ feeds back to inhibit the channels [49,57]. Calcium pumps (PMCAs) present one mechanism for Ca^2+^ removal, and, while there are many genes (23) for calcium pumps (PMCAs) in *Paramecium*, there are only two gene products that are particularly abundant in the cilia [53,55]. Yano and Van Houten found that not only does RNAi for these PMCAs (ptPMCA2a or 2b) prolong backward swimming showing that Ca^2+^ removal is impaired [55], but also these pumps co-immunoprecipitate with the Ca_V_ channels α subunits and are found in the same density fractions of ciliary membrane preparations. Like the Ca_V_ channels, these PMCAs do not appear to rely upon the BBSome for trafficking to the cilia. A summary is presented in Figure 8B.

The Ca_V_ channel story is also particularly interesting in that there are small proteins required for the function of the Ca_V_1,2,3 channels that are exclusive to the ciliary membrane and function in the execution of the action potential. Kung launched a genetic dissection of behavior in *Paramecium* as an approach to identifying the channel proteins that regulate swimming behavior and the action potential. Among selected mutants were the Pawns, named for the chess piece that can move only forward. These cells cannot back up if they encounter an obstacle or when they are in the presence of depolarizing stimuli that open the Ca_V_ channels and initiate the action potential [46]. When the Pawn proteins, A or B, are mutated, the Ca_V_ channels are not found in the cilia. However, because Yano could epitope tag the channels, he and Lodh could show that these small proteins and not the BBSome are involved in the journey of the Ca_V_ channels to the cilia [54,58]. Summary Figure 8B.

The take-away information from *Paramecium* BBSome studies is that ion channels and ancillary proteins that are specific to the cilium can take different paths into the ciliary membrane. K channels depend upon the BBsome and upon specific different BBSome component proteins. The TRP like channel PKD2 also takes a pathway that depends upon the BBSome, but its partner XNTA does not. The Ca_V_ channel is independent of the BBSome, as is the PMCA that seems to be in close proximity with the channel once in the ciliary membrane. The channel requires accessory proteins called PawnA and B to reach the cilium, but the PMCA does not. The channel and pump might be assisted in movement to the cilium by phase separation into a specific membrane fraction.

**Figure 9:**
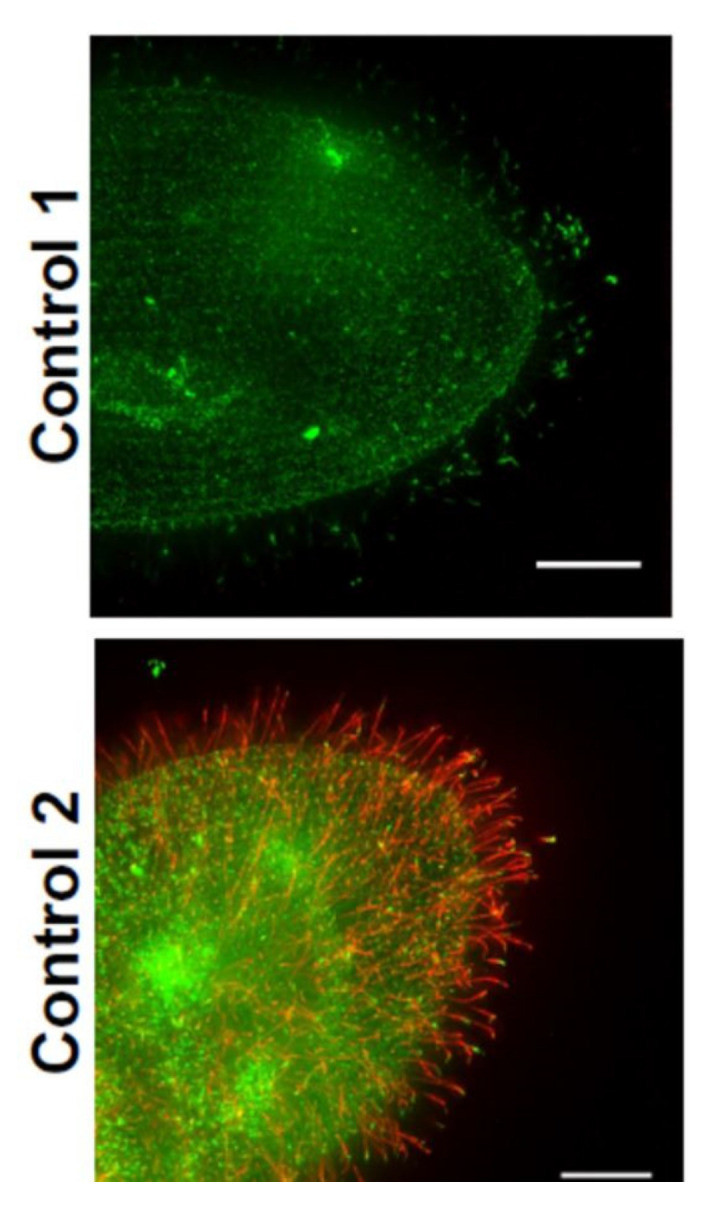

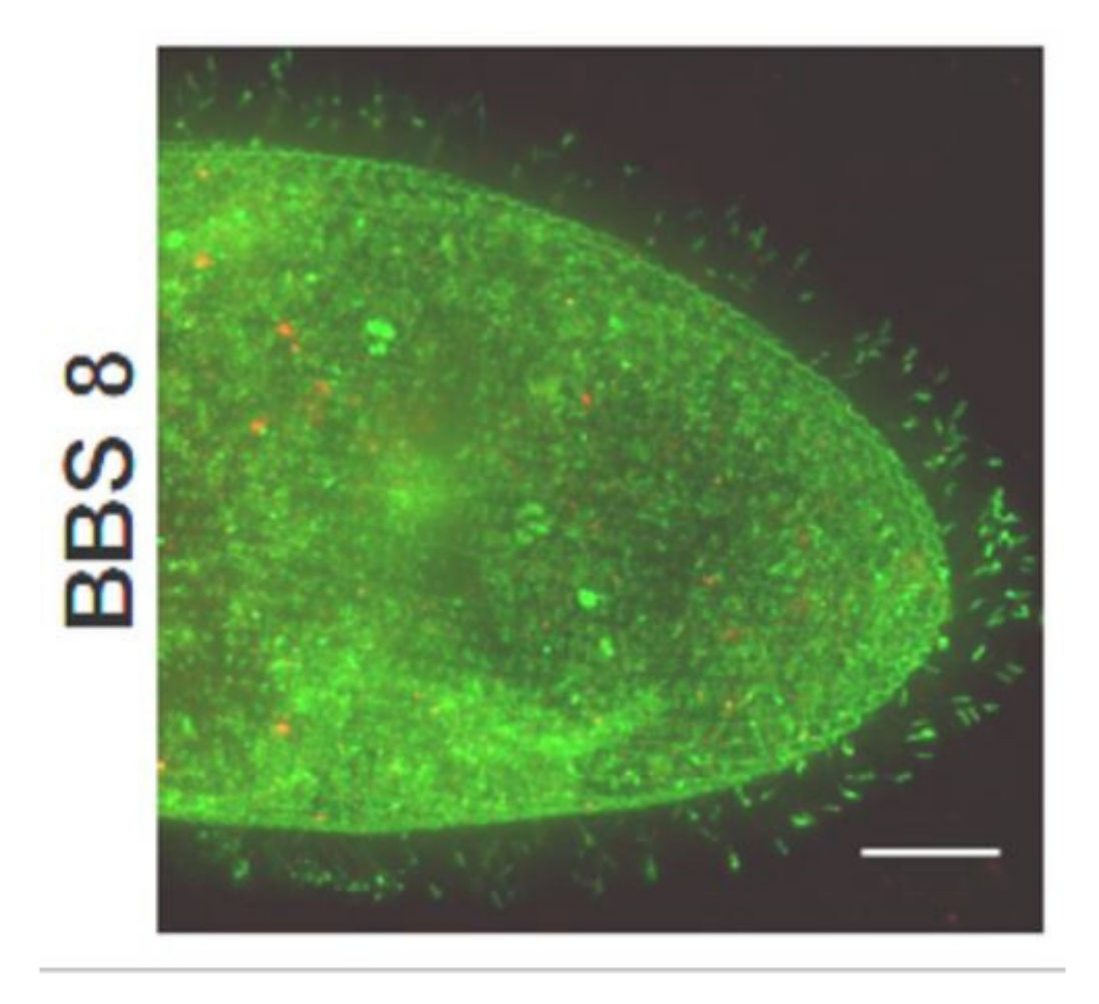
Immunofluorescence of FLAG-SK1a channels and folate chemoreceptor (FBP) a GPI anchored protein with antibodies against the FBP. Note that both are in the cilia, while FBP is in both the cilia and cell membrane. Only the merged images are shown here, but all controls are available [10]. FLAG control is a cell microinjected with FLAG-pPXV vector and fed RNAi empty vector bacteria. FLAG-SK1a control is a cell expressing FLAG-SK1a and fed bacteria with an RNAi empty vector. “BBS8”cells are expressing FLAG-Sk1a channel and are also depleted of BBS8 RNA by RNAi. Cells were immune stained with anti-FLAG (red) and anti-FBP (green) antibodies. These images are from a larger published study with more BBS genes silenced, and with similar results. Note that Sk1a is missing from cilia after silencing BBS8, but the FBP remains in the cilia. Reproduced from [8], with permission.

Another point to be gained from this section on *Paramecium* physiology is that these motile cilia are sensory. They clearly respond to membrane potential and change ciliary beat with potential changes. The cells also respond to chemical cues. They have chemo-receptors for folate (Figure 9), glutamate [59,60], cyclic AMP [61], and other stimuli on their cilia. Attractant stimuli like folate, glutamate, or cAMP hyperpolarize the cell thus causing the cilia to beat faster with fewer avoiding reactions; repellents cause the cilia to reverse power stroke to cause frequent turns and slower swimming [62,63]. The cilia provide the motor response of chemical signaling and also contribute to the sensory transduction through the chemoreceptors which can be on both cilia and cell body. 

## 3. *Paramecium* PKD2 Channel

Polycystin 2 (PKD2) is a non-selective transient receptor potential (TRP) cation channel, which, when mutated or absent, leads to 15% of Autosomal Dominant Polycystic Kidney Disease (ADPKD) cases [64,65]. The other 85% of ADPKD cases mostly arise from mutations in the much larger PKD1 protein, thought to be a mechanoreceptor located in the cilia [66]. This disease is characterized by cystic kidneys that become less functional over time, eventually requiring dialysis and eventually a transplant, and is classified as a ciliopathy. The PKD2 channel is not always located in the cilia, it can also be observed in the endoplasmic reticulum involved in intracellular calcium release through interactions with the ryanodine receptor [67] or IP_3_ receptor [68]. The PKD2 protein commonly interacts with the larger PKD1 protein [69,70,71,72] to facilitate ion entry into a cilium or cell. However, it also forms heteromers with other TRP channels including TRPC1 [72,73,74,75,76], TRPC4 [74,77,78], TRPV4 [74], and homomers [76,79]. In mammalian cells, the PKD2 channel has been shown to be permeable to many different ions, including Ca^2+^, K^+^, Na^+^, Cs^+^, Ba^2+^, and Mg^2+^ [80,81,82]. 

In *Paramecium*, Valentine found that *Pkd2* is the homolog of mammalian PKD2. While the mammalian PKD2-interacting partners listed above do not appear to have homologous genes in *Paramecium,* the Pkd2 protein does have a partner, XntA1 [56]. *XntA1* gene is named *Eccentric* because of abnormal swimming behavior by mutants [83] in Mg^2+^ solutions. Normally, cells have a large Mg^2+^ selective current and swim backward in Mg^2+^ solutions. Xnt1A mutants do not show this behavior due to failure of a Ca^2+^ activated Mg^2+^ channel [84]. XntA protein is small and unrelated to any other known interacting partner of mammalian PKD2. However, it has characteristics of an exchanger protein and is critical for the Mg^2+^ current [85]. The XntA protein is located in the same areas of the cell as Pkd2: the cell membrane and in the cilia [56]. Although the Pkd2 channel requires the BBSome to traffic to the cilia [8], XntA is not totally dependent upon the BBSome (unpublished results in Figure 7). The trafficking of Pkd2 in *Paramecium* also does not require XntA and vice versa [56]. Over expression of Pkd2 rescues the XntA1 mutant phenotype rendering the mutant permeable to Mg^2+^ and able to swim backward in Mg^2+^ [56,83,84,85]. 

Valentine used electrophysiology to show that when XntA1 mutant cells were deciliated, they were permeable to Mg^2+^, suggesting that XntA may regulate the Pkd2 protein in the cilia, but that the channel can act without XntA in the cell membrane [56]. Our studies showed that these two proteins, Pkd2 and XntA, interact at the C-terminus of Pkd2 in both the cilia and the cell membrane, and that most likely, Pkd2 is the Mg^2+^ channel in *Paramecium* and the XntA protein has some type of a stabilization role for Pkd2, and most likely other proteins as well [56]. 

The *Paramecium* Pkd2 and its partner XntA1 broaden the possibilities for interacting partners, functions of these partners, and locations of ciliopathy proteins. 

## 4. Transition Zone

As discussed above, for some proteins meeting up with IFT trains for entry into the cilium can be orchestrated by the BBSome [36,37,38,39]. Other proteins require the BBSome to pass back through the transition zone to exit the cilium [24,25,41]. 

### 4.1. Transition Zone Proteins 

**Figure 10:**
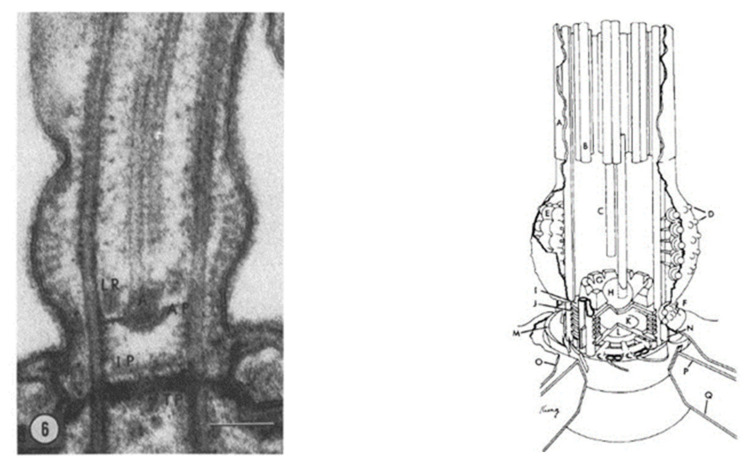
Ultrastructure of the *Paramecium* transition zone (A) cross-section of a *Paramecium* transition zone showing the terminal plate (TP), intermediate plate (IP), and axosomal plate along with the axosome (A) and loosely packed ring (LR). (B) The 3D reconstruction of the *Paramecium* transition zone and beginning of the cilium that is attached. The ciliary membrane (A) is contiguous with the plasma membrane (O). the alveolar sacs that lay just below the outer (P) and inner (Q) membrane. Extending above the transition zone are the central tubules (C), only one of which enters the axosome (H). The peripheral doublets (B) of the cilium also begin above the plaque particles (D) that cover the plaque complex (E). There is loosely packed ring material (G) that surround the axosome that site ajust above the axosomal curved plate (I). The ciliary necklace (F) surrounds the cilium near the rings that connect the peripheral tubes (J). The intermediate plate (K) sits at the center just above the terminal plate (L). Transitional fibers (M) and projections from the peripheral tubules (N) are also shown. Reproduced from [86], with permission.

Defects in the transition zone (TZ) lead to a variety of ciliopathies, including the Meckel-Gruber Syndrome (MKS) and Nephronophtysis (NPHP module) [5]. Note the location of the TZ in Figure 2 and Figure 3, and the modules of MKS and NPHP proteins in this region. The conserved proteins in this highly regulated region help to make up the Y-links that are critical gate keepers for passage of select proteins into and out of the cilium [5,7]. *Paramecium* provided one of the first detailed examinations of this zone and the Y-links using transmission electron microscopy [86] (Figure 10). This TZ has been clearly defined as the area between the terminal plate (closest to the cell), through the intermediate plate and ends at the axosomal plate (furthest from the cell) [86,87]. The terminal plate of the TZ is organized around a central rim that has nine spokes that reach into the gap between the microtubule doublets and contain particular proteins called epiplasmins [88]. The TZ of non-ciliated basal bodies is more collapsed than in ciliated basal bodies [87]. 

*Paramecium* has many of the TZ’s NPHP and MKS proteins coded within their genome (Table 1, Modified from [5,11]). Most of the components in the MKS module are conserved in *Paramecium*, while only NPHP4, also known as POC10, that is a component of the NPHP module is present (Table 1) [5,11]. Through a systematic tagging effort of five of these transition zone proteins, TMEM107, TMEM216, CEP290, RPGRIP1L, and NPHP4, clear TZ localization and proximity to one another was determined as well as information on the involvement of these proteins in the retention or shedding of cilia [11]. The TZ plays an important regulatory role in ciliary content by acting as a ‘gate’ to proteins both entering and exiting the cilia and defective or missing proteins from this zone have been implicated in the aforementioned ciliopathies [5,27]. 

**Figure 11:**
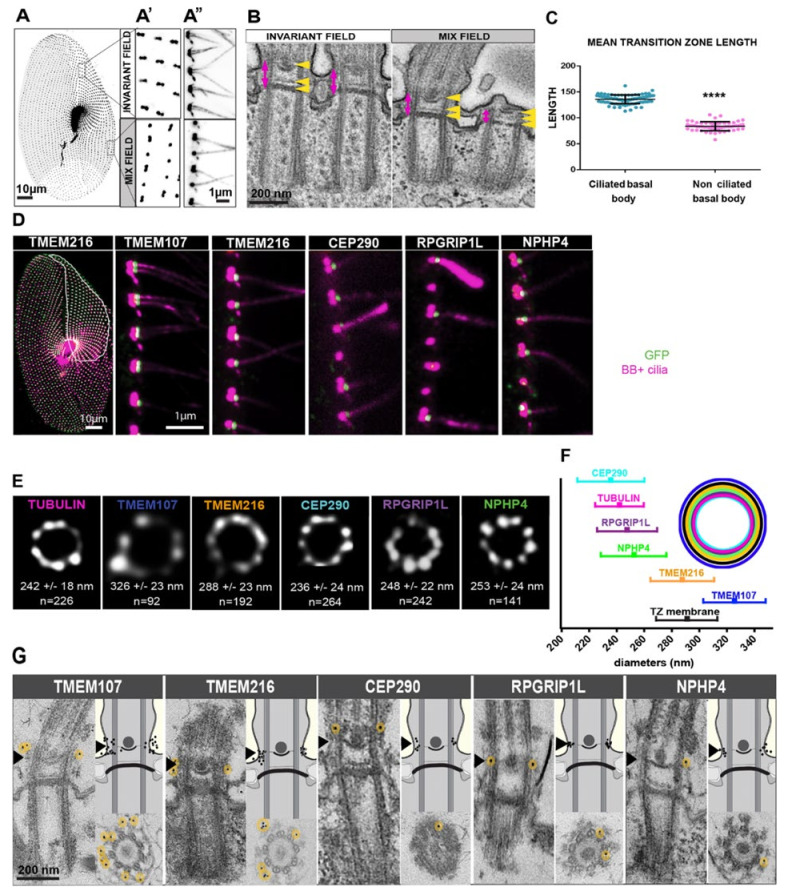
Localization of TZ proteins in *Paramecium.* (**A**) Paramecia labeled by 1D5 to identify the basal bodies showing the invariant field (double basal body units) at the anterior of the cell and the mixed field (both single- and double-basal body units) (**A’**). The antibody also decorates the cilia (**A’’**), scale bars are labeled. Electron microscopy images show longitudinal sections of these basal bodies in the invariant (left) and mixed (right) fields. (**B**) The yellow arrows show the terminal plate (bottom), the intermediate plate (middle) and the axosomal plate (top) present in the TZ (pink vertical double-arrows). Note that the basal body on the far right from the invariant field shows a shorter, more compressed TZ, as this basal body does not have a cilium, scale bars are 200 nm. (**C**) Graphical representation of the mean length of the TZ in ciliated and non-ciliated basal bodies (56 ciliated and 100 non-ciliated basal bodies were measured, **** *p* < 0.001, unpaired 2-tailed *t*-test. (**D**) *Paramecium* expressing one of the five different TZ proteins (TMEM107, TMEM216, CEP290, RPGRIP1L, and NPHP4) tagged with GFP. Basal bodies are also stained (pink, 1D5), invariant zone is highlighted in white on the shown TMEM216-GFP expressing cell. Notice that the GFP-tagged protein is only shown at the distal part of ciliated basal bodies (green staining). Note that NPHP4-GFP can also be seen at the proximal end of the basal body. (**E**) STED images showing the 9-fold symmetrical localization of each protein and the diameter of these rings were measured to produce a graph (**F**) of the mean diameter with standard deviation of these proteins with the top-right showing respect to the basal body and to the ciliary membrane. (**G**) shows a representative TEM image of the localization of the different GFP-tagged proteins. The lower panels are transverse sections of basal bodies at the plane of the axosomal plate. The upper-right panels show the position of gold beads using diagrams. The left panels all show longitudinal views. Although the proteins all have different diameters, they are all found at the axosomal plate (black arrowhead). Image from [11], reproduced with permission.

Through the use of GFP-tagging, immunogold labeling, and stimulated emission depletion microscopy (STED), the positions of *Paramecium* TMEM107, TMEM216, CEP290, RPGRIP1L, and NPHP4 were clearly shown to have a 9-fold symmetry [14]. Both CEP290 and RPGRIP1L are more central in the TZ, closer to the microtubules while NPHP4 localized outside of the microtubule doublets (Figure 11). Both TMEM216 and TMEM107 were more external, closer to the membrane while the recruitment of all were needed for molecular and structural maturation of the TZ [11]. In addition, it was clear from swimming assays that when any of these genes were depleted, both swimming velocity and swimming patterns were abnormal, while basal body positioning remained unchanged [11]. These results suggest that these cells have an abnormal distribution of channels present in or missing from the cilia. Further studies, examining the channel proteins and the use of electrophysiology, could shed light on what channels have had their location altered when these important TZ proteins are depleted. We know that the depletion of some of the proteins (namely, TMEM107 or TMEM216) made cilia more fragile, while the depletion of other proteins (specifically CEP290 or RPGRIP1L) showed that cilia were more resistant to breakage. Because the removal of cilia is a calcium-based process, there is most likely an interruption to the calcium-dependent breakage of cilia. 

### 4.2. TZ continued: Meckel–Gruber Syndrome (MKS) 

NPHP, JBTS, and Meckel-Gruber (MKS) syndromes have in common that they are autosomal recessive ciliopathies associated with cystic kidneys, retinal degeneration, and cerebellar or neural tube malformation [5,7]. While they share some phenotypes, the MKS syndromes are the most severe (see Figure 2, Figure 3 for MKS modules).

**Figure 12:**
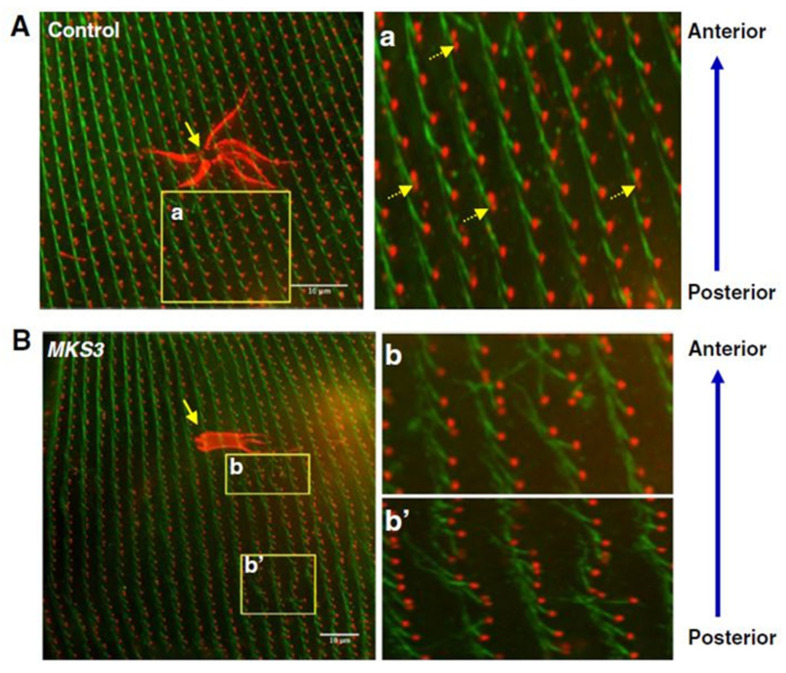
Chaotic orientation of the striated rootlet of *MKS3*-depleted cells. Control cells (**A**) and *MKS3*-depleted cells (**B**) were stained with anti-Glu-α-tubulin (red; basal bodies) and anti-striated rootlets (SRs) (also called anti-kinetodesmal fibers; green). Yellow arrows in (**A**) and (**B**) indicate the contractile vacuoles on the dorsal surface of these cells. SRs project from the basal bodies. In two basal body units, SRs project only from the posterior basal body (**a**; dotted yellow arrows). SRs project toward the anterior of the cell in a highly organized manner along the basal body row (kinety). Cells depleted of *MKS3* show chaotic organization of the SRs, which project in every direction (**b** and **b**’). Scale bar: 10 µm. Reproduced from [12], with permission.

Meckelin (MKS3) family proteins are necessary for the proper localization and formation of cilia. As in other systems, reduction of MKS3 leads to short and missing cilia in *Paramecium* and also a new phenotype of misalignment of longitudinal rows of basal bodies, rotation of the orientation of the basal bodies and their rootlets (work of Valentine and Picariello in the Van Houten lab) [12]. Figure 12 presents that the loss of cilia causes irregular movement of the cilia and loss of metachrony. Rotation of the basal bodies would also cause swimming problems similar to that of the Striated Rootlet example below with cilia power strokes going in random directions.

**Figure 13:**
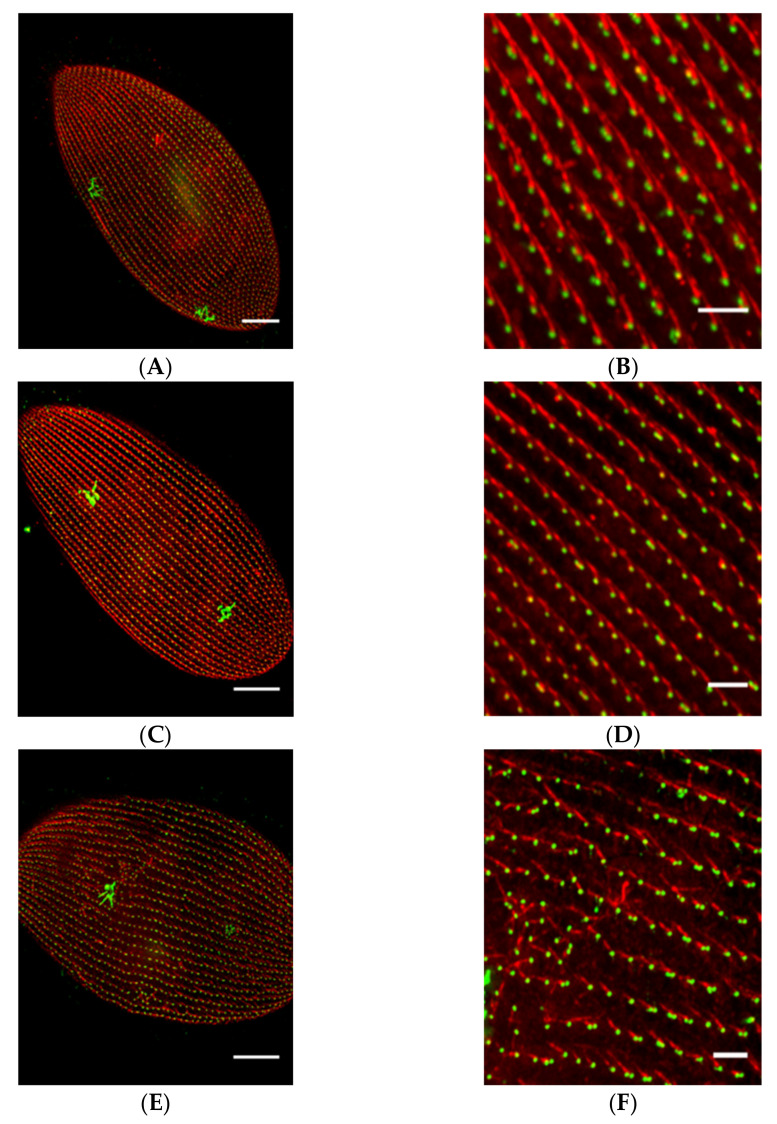
Depletion of MKS5 cause loss of cilia but does not affect the straight basal body rows and Table 1. D5 and anti-SR antibody to visualize the basal body units (green) and SRs (red) respectively on the cell surface. Panels A and B show the basal body row alignment and SRs organization in the control cell. In the control cell basal body rows remain straight and SRs show a highly ordered organization. SR emanates from the basal body unit and extends towards the anterior pole of the cell. Panels C and D show the basal body row alignment and SRs in the MKS5 depleted cells. The phenotype of the cell is similar to the control cell. Panels E and F show the basal body row alignment and SRs in the MKS3 depleted cell. In the MKS3 depleted cell, basal body rows are misaligned and SRs have a disordered organization on the cell surface. Scale bars are 15 µm in (**A**,**C**,**E**) and 3 µm in (**B**,**D**,**F**). Reproduced from [13] with permission.

**Figure 14:**
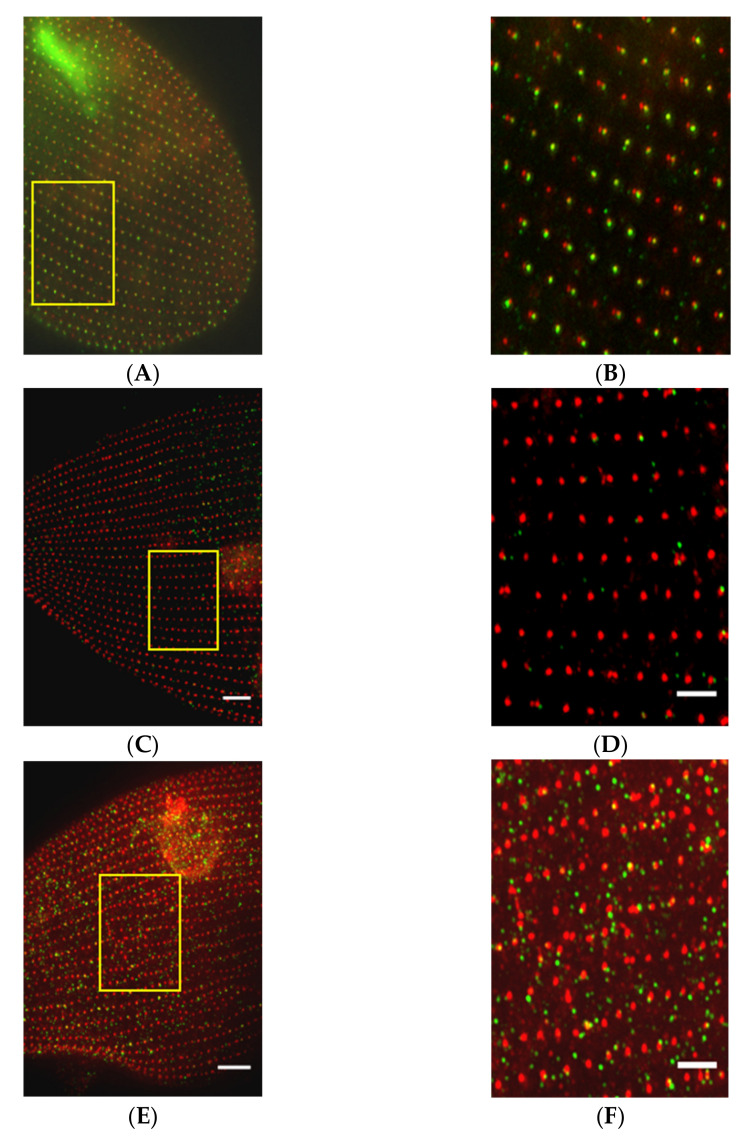
Depletion of MKS5 affects the localization of B9D2 protein in TZ of the basal body. All cells were treated with anti-*Tetrahymena* centrin and anti-GFP antibody to visualize the basal body units (red) and GFP-B9D2 protein in the cell. In all the images, the yellow box highlighted area is enlarged to show the basal body rows and GFP localization in the GFP-B9D2 expressing cell. Panels A and B show the basal body row alignment and GFP-B9D2 localization in the control cell. In the control cell, basal body (red) rows remain straight with GFP-B9D2 protein localization at the TZ of the basal body in a single basal body unit. In two basal body units, only the posterior basal body has the signal for GFP-B9D2 protein. Panels C and D show the basal body row alignment and GFP-B9D2 protein localization in the MKS5 depleted cell. In the MKS5 depleted cell, basal body rows remain straight like the control cell but GFP-B9D2 localization shows a remarkable difference compared to the control cell. Both the single basal body and the posterior basal body of the two basal body unit lack the localization of B9D2 protein in the MKS5 depleted cell. Panels E and F show the basal body row misalignment and localization of B9D2 protein in the MKS3 depleted cell. In the MKS3 depleted cells, cell basal body rows are misaligned and GFP-B9D2 protein shows very dispersed and diffused localization. Scale bars are 10 µm in (**A**,**C**,**E**) and 3 µm in (**B**,**D**,**F**). Reproduced with permission from [13].

Likewise, MKS5 depletion by RNAi (Figure 13) causes loss of cilia, but does not affect the straight basal body rows and orientation of basal body rootlets (Figure 13C,D) as does MKS3 (Figure 13E,F) (work of Nabi, Van Houten lab [13]). Transition Zone protein, B9D2, is missing from MKS5 depleted cells’ basal bodies (Figure 14) [13]. In contrast, MKS3 depleted cells show basal bodies out of alignment, which is expected, given the chaotic orientation of B9D2 protein.

RNAi for another homolog for MKS1 leads to reduced numbers of cilia and, hence, to abnormal swimming patterns [89]. As with MKS3 and 5, these abnormalities from reduced MKS1 are due to orientation and structural problems of the cilia, not due to their bioelectric control. Nonetheless highly disrupted swimming patterns result. 

**Figure 15:**
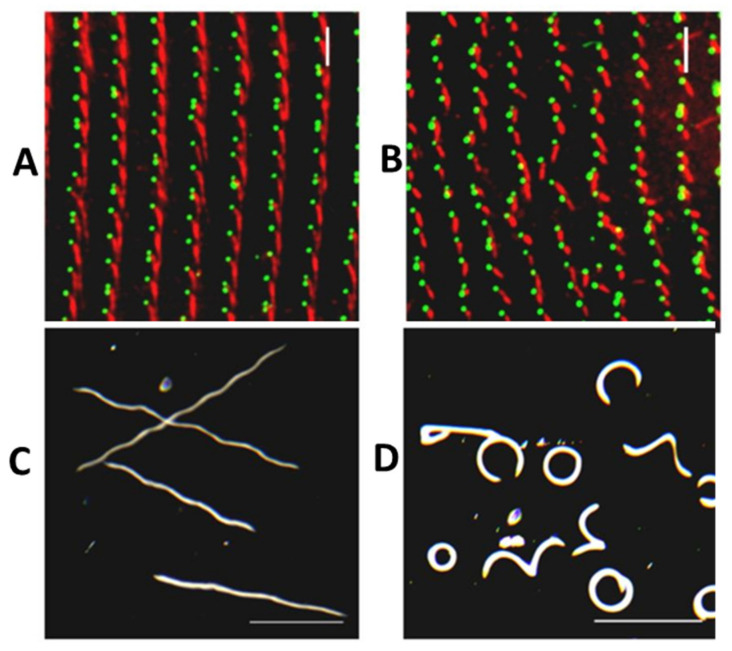
Striated Rootlet Disruption Control (**A**,**C**) and Structural Group 1 Depleted Cells (**B**,**D**). (**A**,**C**) Basal body row alignment and SR appearance in control cells (**A**) or cells with SR Structural Group 2 depleted by RNAi. Basal bodies are green (1D5 antibody) and SRs are red (anti-SR antibody). Note that in the Structural Group 2 SR RNAi treated cells, SRs are shorter and pointing in directions out of alignment with the basal body rows as in the Control Cell (**A**). (**B**,**D**) Swimming patterns of cells taken by darkfield microscopy. Shown here are Control (**B**) and Structural Group-1 depleted (by RNAi) cells (**D**). Scale bar is 1 mm. Reproduced from [90], with permission.

The use of *Paramecium* to study MKS gene function highlights the role of behavioral phenotypes in broadcasting the defects due to the silencing of a gene, for example. Because there are so many cilia on the cell surface in very regular patterns and rows, and because these cilia normally move the cell in well described trajectories, any deviation from normal pattern or swimming is very obvious and alerts the observer to a change in ciliary function. (See also Figure 15 below.)

## 5. Basal Body Positioning and Anchoring

The docking and positioning of basal bodies are critical for proper ciliary development and maintenance and defects in these two processes can lead to ciliopathies. Both primary and motile cilia require a basal body to develop. Basal bodies, the required anchors for cilia (or flagella) to develop, are almost identical to centrioles, differing only in their appendages that anchor the basal bodies below the surface [91]. There are multiple ways basal bodies can develop. Briefly, in mammalian cells, centrioles will migrate toward the cell surface and generate a cilium [91,92,93]. In the case of multi-ciliated epithelial cells, the centriole must replicate itself many times before approaching the cell membrane to generate cilia [91]. In ciliates, including *Paramecium*, this process is slightly different. Although the structures of centrioles and basal bodies are mostly the same, the basal bodies in these ciliated models are not formed from centrioles [94,95]. Instead, the numerous basal bodies located in these model organisms develop adjacent to and derived from a previous basal body, the mother basal body [96,97,98]. The basal bodies in the main body of the cell are organized in longitudinal rows coursing from the anterior to the posterior of the cell. The abundance of basal bodies in these ciliates, up to 4000 per cell in *Paramecium* [96], making them an attractive organism for the study the components of these ciliary anchors and of basal body dysfunction.

Basal body structure was investigated nearly 50 years ago using transmission electron microscopy using the rhesus monkey oviduct and primary cilia (9+0 pattern) that lack a central pair of microtubules [99]. Cilia formation and structure was examined from fibroblasts and muscle cells collected from a wide array of organisms to examine how these organelles formed [92]. Paramecia provided one of the first examinations of the structure of motile cilia and the TZ, showing in amazing detail the structure and organization of these areas [86] (Figure 10). The transition zone is clearly defined and analyses of these structures continue to be used to support growing use of *Paramecium* to better understand basal body structure, protein localization, and connections to ciliopathies.

Basal bodies are key to the organization and positioning of cilia and, not surprisingly, many proteins contribute to the development, anchoring, and tilting up of basal bodies. Defects in or the absence of some basal body proteins have shown direct links to ciliopathies, including nephronophthisis (a recessive cystic kidney disease) [100,101] and orofaciodigital syndrome (OFD) [102]. Understanding what proteins are conserved as well as identifying unique proteins and their function can provide important insights into these ciliopathies as well as conserved proteins among ciliates and other eukaryotes. 

In *Paramecium*, the centrin proteins have been studied extensively by Beisson and others for their association with basal bodies and cilia. Centrin, a calcium-binding EF-hand protein, localizes to microtubule organizing centers and is highly conserved in multiple species [103]. *Paramecium* have 30 centrin genes that separate based on homology into several subfamilies [104]. Based on homology and antibody staining, *Paramecium* centrin 2 (PtCen2) is most closely related to human centrin 2 and *Paramecium* centrin 3 (PtCen3) is most closely related to human centrin 3 [104]. By using GFP-tagging along with human centrin antibodies, PtCen2 was found to be localized to the basal body shaft, the basal body lumen, and the microtubules. PtCen3 localized to a space where the two basal bodies connect. Targeted depletion and over-expression of these basal body components showed that loss of PtCen2 or PtCen3 was lethal [104]. Because of the understanding of *Paramecium’s* basal body duplication process and by studying the cells early in their depletion treatment (these cells divide approximately every 4 to 6 h), researchers were able to observe that loss of PtCen2 affected the position and microtubule shaft stability while depletion of PtCen3 disrupted the final positioning of the basal body where the daughter basal body does not separate from the mother basal body appropriately [104]. These results showed for the first time in detail the importance of centrins for basal bodies, and not just mitotic spindles.

Centrin 2 in *Paramecium* is crucial for the recruitment of other proteins, such as FOR20, to the basal body [87,104]. The FOR20 gene is a member of the FOP-related proteins with a size of 20kD (hence, FOR20) and is highly conserved in most ciliated cells. FOR20 is distantly related to the OFD1 (oral-facial-digital 1) protein [105]. The functional role of FOR20 has been examined in non-motile cilia (9+0 axonemal configuration) in REP1 cells resulting in stunted cilia when FOR20 was depleted [105]. Research done using *Paramecium* provided detailed localization information for the protein in the transition zone of all basal bodies, including those without cilia [87]. Without FOR20, basal bodies were unable to dock at the cell surface and unable to mature, similar to loss of Centrin 2 [87].

**Figure 16:**
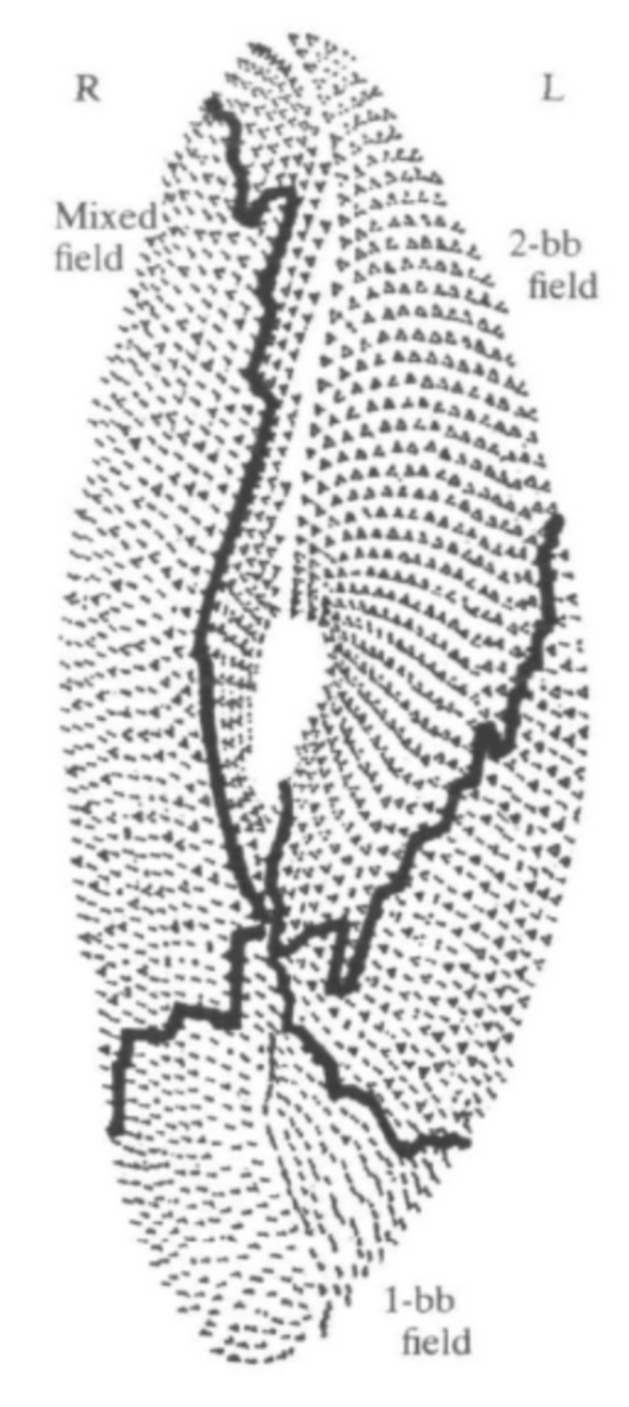
A camera lucida drawing shows the ventral surface of *Paramecium*. The anterior two basal body field (2-bb field), or invariant zone, contains all two basal body units, each with a cilium arising. The mixed field wraps around the cell and contains cortical units with either a single- or double-basal body. In this region, only the posterior of the basal bodies has a cilium arising from it. The single basal body field (1-bb field) at the posterior of the cell contains cortical units with only one basal body, each with a cilium arising (Image from [96], reviewed in [98]). Reproduced with permission.

Additional proteins have been identified for their role in basal body positioning and anchoring that would not have been possible without the information learned from studies of the cortical arrangement and duplication of *Paramecium* basal bodies [96,106]. The surface of *Paramecium* is divided into three regions, or zones, based on the types of surface cortical units that exist there (Figure 16). The anterior of the cell contains the invariant zone, where all cortical units contain two ciliated basal bodies. The posterior of the cell contains only single basal body units with a single cilium. The middle portion of the cell is referred to as the mixed field, where there are both single- and double-basal body cortical units. In this mixed field, it is only the posterior basal body that is ciliated [96,98]. 

*Paramecium* has provided vital information for gene products involved in the process of basal body positioning and polarity (reviewed in [98,107]). One gene product, OFD1 (oro-facial-digital syndrome 1), is important for basal body anchoring for primary cilia as well as the attachment of distal basal body appendages in mammalian cells [108]. The second, VFL3 variable flagellar number 3 (VFL3), plays a not well-understood role in multi-ciliated cells. However, roles the OFD1 and VFL3 gene products play in basal body polarity were uncovered using *Paramecium* as a model [109]. GFP-tagging of OFD1 showed the protein localizes at the proximal part of the basal body very early in development, similar to the immunofluorescence patterns shown by FOR20 and centrin 2, but OFD1 did not require centrin 2 for proper localization [87,109]. Similar to the observations in mammalian centrioles, there was an inter-dependence of FOR20 and OFD1 on each other for localization [110]. Depletion of VFL3 in *Paramecium* revealed basal bodies with missing rootlets, some with too many, and basal bodies developing in incorrect locations [109]. The depletion of OFD1 in *Paramecium* leads to a defective transition zone and, in turn, an inability of basal bodies to properly anchor at the cell surface, in agreement with studies in mammalian primary cilia [108]. VFL3 is clearly required for basal body polarity and proper rootlet attachment. These novel findings about the importance of OFD1 and VFL3 in motile cilia would have been far more difficult had it not been the extensive knowledge and understanding of basal body duplication, anchoring, and replication in *Paramecium*. 

## 6. Rootlets—Roles in Basal Body Positioning

Cilia, including those of *Paramecium*, have rootlet structures at their basal body base, which help to resist the torque [111,112] from the ciliary beat and assist in maintaining the appropriate spacing for metachronal beating among multiple cilia [90,112]. A mammalian protein, Rootletin, is a component of the ciliary rootlet and appears to have several roles. One is to interact with the centrosome and function in the control of ciliogenesis and control of the number and timing of cilia [113]. Another is the maintenance of retinal receptor cell. Photoreceptor cells without Rootletin have no ciliary rootlet and deteriorate over time. Sensory cells lose their rootlets and mechanosensory function [114,115]. Without rootletin lung mucociliary clearance is insufficient [116,117].

**Figure 17:**
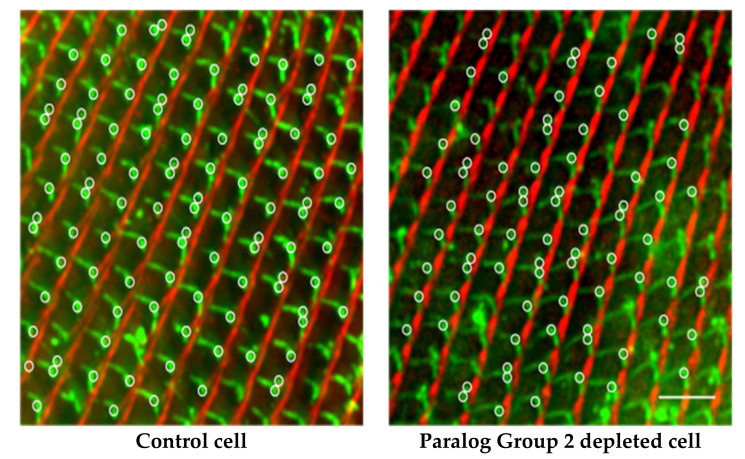

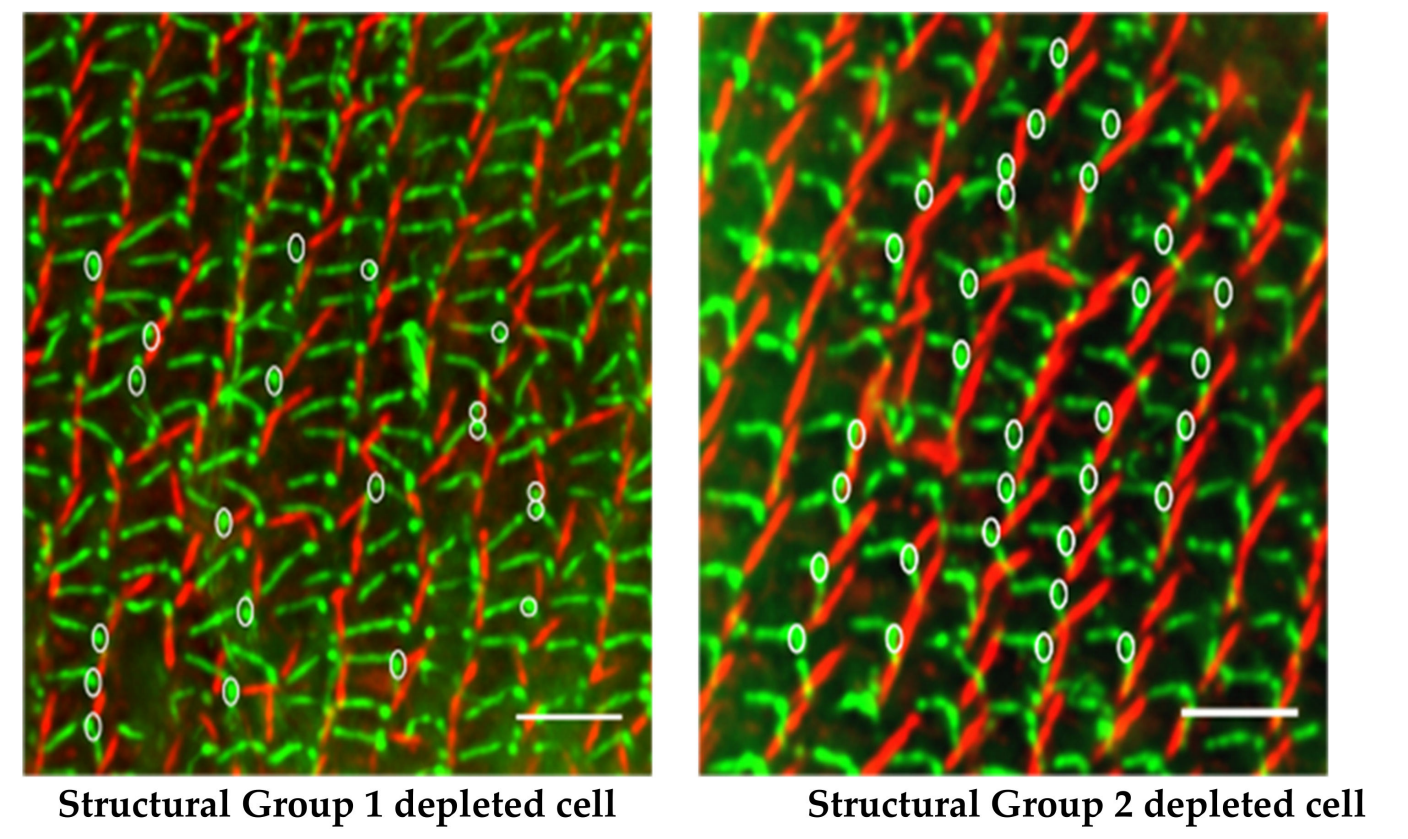
Striated rootlet structure and alignment disrupted by RNAi. Depletion of SR proteins leaves the angles between the rootlets unaffected. Images show staining of all three rootlets (TR, green; PR green; SR, red). TR and PR and stain green with anti-acetylated tubulin. Basal body stains green with 1D5. SR is stained red with anti-SR antibodies (courtesy of J. Beisson). TR stretches across the basal body row; PR stretches toward the posterior; SR points toward the anterior, in alignment with the basal body row. Scale bars 3 µm. White circles denote the position of basal bodies with all three rootlets visible that were used in measuring the angles between rootlets. At least 100 basal bodies with rootlets were examined for each condition. Pick a white circle in the first two panels and see that there clearly are two green rootlets stretching out from the basal body and a red rootlet in line with the row of basal bodies. The control image shows the three rootlets attached to the basal body, with the SRs in red joining to form a clear line pointing toward the anterior of the cell. No change was expected for Paralog Group 2 depleted cells, but Structural Group depleted cells were expected to show misalignments, as they clearly do here. When the angles between the three rootlets were measured, there was no change in the angles despite what looks like a rotation of the basal body and rootlets out of alignment with their row. Reproduced from [90], with permission.

In *P. tetraurelia*, there are two microtubule-based rootlets (transverse (TR) and postciliary (PC)) that arise from triplet microtubules of the basal body, maintaining a fixed angle between them. A non-tubulin-based rootlet, the Striated Rootlet (also called kinetodesmal fiber), likewise arises from the basal body in a fixed position relative to the other rootlets. The Striated Rootlet (SR) is composed of SF assemblin proteins that organize into these very large structures with striations (see [90] for a review). The array of SF assemblin proteins is very large (30) but Nabi in the Van Houten lab found that it can be organized into 13 Paralog groups and further into five Structural Groups according to their amino acid sequences. Silencing individual SR genes or Paralog Groups has no effect on the surface organization, but silencing any of the Structural Groups triggers the misalignment of rows of basal bodies: SRs pointing in wrong directions, shorter SRs missing their striations, and distorted cortical units of the surface. The three rootlets (SR, TR, and PC) maintain their normal angles (Figure 17), but the basal bodies are not properly organized into rows, causing their cilia to beat out of synchrony and cells to swim in highly abnormal paths (Figure 15). See [118] for a review of mouse basal bodies and rootlets. Further, for brief reviews of the function of rootlets, see [90,111,115,119,120].

## 7. IFT Components

Proteins are not synthesized in the cilia. Proteins must be transported to the base of the cilium, through the transition zone (discussed above) that acts as a gateway, and up into the cilium to build and maintain the structure. The contributions of studies of the *Paramecium* transition zones are numerous and are highlighted next.

Intraflagellar Transport (IFT) was first identified in the 1990s and has since been extensively examined because of the importance of trafficking cargo up and down the cilium [121,122,123]. IFT has two main complexes, B and A, that are connected to a kinesin-II anterograde motor, that moves cargo from the base of the cilium to the tip, and a dynein retrograde motor that moves cargo from the tip of the cilium back to its base (Figure 2) [5,121,124]. *Paramecium’s* genome has homologues of many genes for these IFT components and RNAi depletion studies have shed light on the cargo being transported as well as changes in the cilia structure. 

One of the first IFT components linked to ciliary defects and ciliopathies was IFT88, a component of Complex B and important for anterograde transport [26,125]. In mice, IFT88 is homologous to the kidney disease gene, Tg737. When Tg737 is defective in these mammals, the kidneys are filled with cysts and the primary cilia are much shorter than in their wild-type littermates, dying a few days after birth, a phenotype consistent with autosomal recessive polycystic kidney disease [125,126]. Upon IFT88 depletion in *Paramecium*, the cells were nearly bald and had delayed cell division and slow swimming [12,17]. Van Houten’s group used the depletion of IFT88 in *Paramecium* provides a reliable positive control for RNAi, knowing that depletion of IFT88 would lead to short or missing cilia and an easily observed surface.

Other studies of Complex B components in *Paramecium* showed results similar to those above when depleted from *Paramecium.* In general, depletion of the Complex B components results in missing cilia and behavioral changes in *Paramecium*, without the lethality observed in mammalian cells. Trafficking of proteins to the cilium for intraflagellar transport in the cilium can be compromised with depletion of IFTB complex proteins. For example, when the Cohen group depleted Complex B IFT172 gene product was depleted, IFT46 was prevented from entering the cilia [9]. The depletion of IFT80 in *Paramecium* showed similar results in that there were fewer cilia, but interestingly, those cilia that remained were full-length [9]. 

The depletion of another B complex component, IFT38/40 (also known as Cluap1 (clusterin associated protein 1), qilin, or FAP22), show that this protein is essential for proper cilia assembly and normal kidney development in mice [127]. In the Valentine lab, the depletion of the B complex component, IFT38/40, showed significantly slower swimming speeds in *Paramecium* [16].

Peripheral Complex B proteins outside the cilium also exist in *Paramecium* and interact with nuclear proteins at certain points during the cell cycle. The IFT57 genes in *Paramecium* have maintained dual roles. There are two pairs of genes, and depletion of any of the IFT57 gene products leads to short and missing cilia [128]. However, one pair of the IFT57 genes localize at the macronucleus and, when depleted, the macronucleus cannot generate a new macronucleus during a rearrangement process called autogamy. These results suggest IFT57 in *P. tetraurelia* has maintained a role in interacting with nuclear proteins at certain time points in the cell cycle while also having a role in ciliogenesis [128].

Complex A components are responsible for retrograde transport and have been associated with ciliopathies. Mutations in a Complex A component IFT140 led to Conorenal dysplasia or syndrome (CRS), also known as Mainzer–Saldino Syndrome (MSS) in humans [129]. This ciliopathy is autosomal recessive, characterized by phalangeal cone-shaped epiphyses, chronic renal disease, retinal dystrophy, and femur abnormalities [130]. Most recently, two novel IFT140 defects have been linked to male sperm abnormalities and infertility [131]. 

Complex A proteins have also been examined using *Paramecium*. Depletion of IFT139, a Complex A protein important for retrograde transport, showed an accumulation of IFT46-GFP at the ciliary tips [9], as the product was unable to return to the cell. Valentine’s group found that the depletion of IFT gene IFT140 from *Paramecium* showed dramatic slowing of the swimming speed. The depleted cells were also much slower to grow and died after 48 h of feeding RNAi bacteria [18]. 

In general, the *Paramecium* model confirms what has been found in mammals with defects in Complex A or B proteins. The utility of *Paramecium* here is the rapid identification of even subtle ciliary presence or beating defects. Follow up using electrophysiology could provide insight into the identity of channels that are affected by the depletion of IFT genes.

## 8. Primary Ciliary Dyskinesia

We have discussed what happens when primary cilia fail to signal whether from failure to move critical proteins into their ciliary membrane (e.g., BBSyndrome) or failure to form a cilium at all (e.g., kidney cells in PKD). When motile cilia fail, the resulting ciliopathy is called Primary Ciliary Dyskinesia (PCD). In the cases of PCD, cilia must not only be present, but they must also be motile for normal development [6,132,133,134,135]. For example, embryonic nodal cilia must beat or spin and generate a leftward fluid flow that is detected by immotile cilia in the node and transduced into the signals that cause left–right laterality of the body. These nodal cilia must beat correctly or conditions such as *sinus inversus* arise. We depend upon motile cilia in our respiratory tracts, ependymal cells of the brain ventricles and spinal canal, fallopian tubes, and male spermatozoa. Afzelius has categorized eight types of cilia that must function to avert PCD [132]. In some cases, the cilia propel mucous to protect our respiratory tracts or propel other fluids as in the spinal cord. They move cells as in the case of spermatozoa. Therefore, PCD is characterized by chronic respiratory infections laterality defects and infertility among others, all of which can be traced back to failure of cilia to beat properly.

Figure 4 depicts a cross-section of a motile cilium, showing the component parts that work to make the cilium bend in a rhythmic way with power strokes and return strokes from different patterns of sliding of the microtubule doublets. Earlier in this review, we considered the ciliary ion channels and also trafficking processes that contribute to the assembly and appropriate function of cilia. Here we are focusing more on the physical parts and assembly for motility. Considering that all aspects of cilium assembly, structure, and function are potential points of failure that can produce PCD, the number of proteins and genes identified in the phenotype of PCD is very large. Proteins that have been associated with PCD include outer and inner dynein arms, radial spokes, nexin links, and transition zone components [133]. Also included are cytoplasmic proteins necessary for pre-assembly or assembly of cilia or trafficking of proteins to and past the transition zone diffusion barrier (see [133,134,135] for tables of genes/proteins and their functions in PCD). There are at least 40 PCD-associated genes [133]. 

An important resource for PCD research is the enormous detail available through ultrastructure studies including those on *Paramecium.* Jurand and Selman produced a treasure trove of ultrastructure details of *Paramecium* cilia and their arrangement on the cortex [136]. A legacy of Richard Allen is his extraordinary image collection of *Paramecium* https://www6.pbrc.hawaii.edu/allen/, accessed on 16 September 2021. The analysis of cilia by Satir and Hamasaki showed that the mechanism ciliary frequency and swimming speed increases induced by cyclic AMP correlated with the phosphorylation of an axonemal 22S dynein [137,138]. Details of the transition zone of the *Paramecium* cilium were revealed by Dute and Kung (Figure 10) [86]. Rotation and twist of the central pair of microtubules during the ciliary stroke was described in Kung and Omoto’s fastidious studies of *Paramecium* cilia [139,140]]. Of course, the long history of ultrastructural analysis of *Paramecium*, including its cilia, has provided an expansive library of the components that need to be in place for proper ciliary function and to avoid problems with swimming and, in humans, PCD [141,142]). The montage of images of the transition zone in Figure 11 shows the quality and variety of technologies, including STED super-resolution microscopy (and tomography not shown) used to gather fine structural details [11]. 

As a demonstration of the contributions that can be made with *Paramecium* research to PCD, there is one article in particular from the Tassin lab on *TTC12*, a gene that is associated with PCD that has homologs in *Paramecium* [14]. *TTC12* codes for a cytoplasmic protein, for ciliary assembly in PCD patients’ cells. There are two phenotypes in patient respiratory and sperm cells: both outer dynein arms and inner dynein arms (ODAs and IDAs) were affected in sperm, but only IDAs were affected in respiratory cilia. In contrast, *Paramecium* depleted of *TTC12* showed the sperm phenotype of affecting both kinds of dynein, providing a way to model the effects of *TTC12* in sperm.

*Paramecium* gene *C11orf70* when silenced leads to the loss of IDA and ODA causing reduced ciliary beating and swimming speed, again showing the potential for a model for IFT transport of ciliary structural protein cargo and PCD [15].

## 9. Limitations as a Model

The *Paramecium* species that we have primarily highlighted here are *P. tetraurelia* and *P. caudatum*. Both are relatively large cells (~150 µm for *P. tetraurelia* and ~300 µm for *P. caudatum*) covered with 4000 or so cilia. While they are complete organisms contained in a single cell, they cannot duplicate the cell types found in a metazoan, thus limiting their application to ciliopathies. They also do not appear to have Hedgehog cilia-dependent signaling, which is so critical in mammalian development [143]. Paramecia are indeed sensitive to mechanical stimulation, but their cilia, even their long caudal cilia, do not appear to be the organelles responsible for this sensitivity [43]. This distinguishes them from primary kidney tubule cilia that must signal mechanical stimulation to avoid kidney cysts [67,77,144]. 

On the other side of the ledger board is the large amount of ciliary material that can be harvested from paramecia, which provides an enormous resource for biochemistry that cannot be easily carried out on primary cilia with one per cell. 

We hope to have convinced you that motile cilia are sensory [23,145] and that *Paramecium* cilia are not to be discounted for their motility.

## 10. Conclusions

For many decades, research on *Paramecium* has contributed and continues to contribute important findings about cell motility and cilia as well as genomics and cell biology [146]. (We had to be selective about what we included in this review, and our apologies to those whose fine research we were not able to include.) These organisms present some practical advantages in the study of cilia, and, because of conservation of many proteins in the development and function of cilia, *Paramecium* research can explore very close gene homologs and homologous structures. This conservation allows studies on *Paramecium* to have relevance for ciliopathies. There are some lessons to be learned about ciliary ion channels, for example, which are most efficiently studied in the *Paramecium* system. 

There are other polycystin proteins in addition to PKD2 and pKD1. Through patch clamping of cilia, Delling and Clapham demonstrate that heteromeric PKD1L1 and PKD2L1 form the main Ca^2+^ channel of primary cilia [147,148]. We have focused on the roles of PKD2 and PKD1 because we found no evidence of PKD1L1 or PKD2L1 proteins in *Paramecium*.

Last, the cilia data base resource created for the cilia community, built by the *Paramecium* resources in Gif-sur-Yvette, can be found on (http://cildb.i2bc.paris-saclay.fr/, accessed on 16 September 2021).

## Figures and Tables

**Table 2:** Research Methods Used to Study Cilia Genes in *Paramecium*.

Method	Application(s)	
RNA interference (RNAi)	This effective method allows researchers to develop knock-down versions of *Paramecium* to examine cells depleted in targeted genes. Off-target analysis helps to ensure that the sequence used to generate the RNAi plasmid will not deplete other genes products or if similar/related genes, such as paralogues, are targeted. Reverse transcriptase PCR or Real Time PCR can also be used to determine the amount of depletion of targeted genes compared to a ubiquitously expressed gene, such as calmodulin or compared to the depletion of a nonessential gene, such as nd7.	
Epitope Tagging	Numerous epitope tags (Flag, GFP, HA, etc.) are available and can be expressed at the N- or C- terminus of a protein. Upon injection into the macronucleus or by using electroporation, these genes can be expressed by the cells and the epitope localized using fluorescent microscopy or used in other methods.	
Immunoprecipitation (IP)	*Paramecium* membranes are easily collected and importantly for studies of cilia, the cilia can easily be biochemically separated from the cell body and collected. By separating the cilia from the cell body, the membranes can be studied altogether or separately. The cilia membrane can also be removed from the axoneme through extensive vortexing and high centrifugation. Solubilization of the membranes can release soluble proteins for IP.	
LC-MS/MS Analysis	The use of mass spectrometry has been instrumental in the identification of potential interacting partners as well as examining the proteins present in the ciliary membrane. This approach can also be used to examine changes in the ciliary proteins when certain gene products are depleted using RNAi.	
Microscopy	Numerous forms of microscopy can be used to study *Paramecium* and their cilia:	
	Fluorescence	Fluorescence microscopy can be used to examine cells expressing epitope tagged genes for the location of their products. These microscopes can, with great detail, provide information on the location of these different gene products within the cell. The expressing cells can also be used for RNAi to examine changes in the location of the expressed proteins.
	SEM	Scanning electron microscopy (SEM) can be used to closely examine the surface of the cilia or the cell surface for changes in the cilia shape or structure.
	TEM	Transmission electron microscopy (TEM) has provided important details on the structure of cilia and the transition zone. It is also helpful for exact localization studies of proteins with immunogold labeling.
	STED	Stimulated Emission Depletion (STED) Microscopy can provide very detailed information on the spatial location of expressed proteins with epitope tags.
Swimming Assays	*Paramecium* change their swimming behavior if channels of the cilia are missing or their abundance is altered. Changes in the length of time cells spend swimming backward in varying solutions after depletion (RNAi) or over-expression of different genes can shed light on channels or other proteins that may be missing or non-functional in the cilia.	
Motion Analysis	The use of imaging where the length of the swimming track of the cells can be examined. Cells are illuminated from the side in a dark space and are imaged over a period of time, producing a swimming trace that can be measured to determine speed or can be used to analyze the number of turns. This approach can be used to assess changes in the swimming speed of cells in different solutions and also can be paired with methods, such as electrophysiology, to infer conductance defects.	
Electrophysiology	Electrophysiology has long been used in *Paramecium* to show changes in membrane potential as well as differences in the membrane conductances of cells. Combined with RNAi or by examining deciliated cells, the different contributions of the cell and ciliary membrane, with and without different proteins, can be examined. The use of electrophysiology has been able to elucidate the differences in the function and contributions of channels that are located in both the cell and ciliary membranes of *Paramecium*, specifically Pkd2. Electrophysiology has long been used in *Paramecium* to show changes in membrane potential as well as differences in the membrane conductances of cells.	

## Data Availability

This is a review article with data that are made available through the primary sources.
